# Disentangling the relative effects of bushmeat availability on human nutrition in central Africa

**DOI:** 10.1038/srep08168

**Published:** 2015-02-02

**Authors:** Julia E. Fa, Jesús Olivero, Raimundo Real, Miguel A. Farfán, Ana L. Márquez, J. Mario Vargas, Stefan Ziegler, Martin Wegmann, David Brown, Barrie Margetts, Robert Nasi

**Affiliations:** 1 Division of Biology, ICCS, Imperial College London, Ascot, SL5 7PY UK; 2 Grupo de Biogeografía, Diversidad y Conservación, Universidad de Málaga, 29071 Málaga, Spain; 3 WWF Germany, 10117 Berlin, Germany; 8 Department of Ecology and Evolution, University Frankfurt, 60438 Frankfurt on Main, Germany; 4 DLR Berlin, German Aerospace Center (DLR), 82234 Wessling, Germany; 9 Department of Remote Sensing, Department of Geography and Geology, University of Würzburg, 97074 Würzburg, Germany; 5 School of Anthropology and Museum Ethnography, University of Oxford, 51/53 Banbury Road, Oxford, OX2 6PE UK; 6 Faculty of Medicine, University of Southampton, Southampton, SO16 6YD UK; 7 Consultative Group on International Agricultural Research (CGIAR), CIFOR Headquarters, Bogor, 16115 Indonesia

**Keywords:** Sustainability, Tropical ecology

## Abstract

We studied links between human malnutrition and wild meat availability within the Rainforest Biotic Zone in central Africa. We distinguished two distinct hunted mammalian diversity distributions, one in the rainforest areas (Deep Rainforest Diversity, DRD) containing taxa of lower hunting sustainability, the other in the northern rainforest-savanna mosaic, with species of greater hunting potential (Marginal Rainforest Diversity, MRD). Wild meat availability, assessed by standing crop mammalian biomass, was greater in MRD than in DRD areas. Predicted bushmeat extraction was also higher in MRD areas. Despite this, stunting of children, a measure of human malnutrition, was greater in MRD areas. Structural equation modeling identified that, in MRD areas, mammal diversity fell away from urban areas, but proximity to these positively influenced higher stunting incidence. In DRD areas, remoteness and distance from dense human settlements and infrastructures explained lower stunting levels. Moreover, stunting was higher away from protected areas. Our results suggest that in MRD areas, forest wildlife rational use for better human nutrition is possible. By contrast, the relatively low human populations in DRD areas currently offer abundant opportunities for the continued protection of more vulnerable mammals and allow dietary needs of local populations to be met.

**Electronic supplementary material:**

The online version of this article (doi:10.1038/srep08168) contains supplementary material, which is available to authorized users.

## Introduction

In Africa's Congo Basin, people eat an estimated five million tons of bushmeat per year^1,2^ and there is evidence that bushmeat is an important source of many nutrients (especially protein, B vitamins, iron and zinc) for both rural and urban households throughout Africa^2^. However, the magnitude of exploitation and consumption, varies between countries and regions, determined primarily by its availability and influenced by such factors as governmental controls on hunting, socio-economic status and cultural prohibitions. In areas where wildlife still exists people collect, hunt, purchase and eat bushmeat. Some people depend on bushmeat because they have no other source of meat or cannot afford alternative sources; others eat bushmeat as a matter of preference or as a luxury item/delicacy for special occasions. The reality in central Africa is that, for the greater majority of rural people, bushmeat represents a vital dietary item for reasons dictated by lack of alternate sources, financial limitations, preferences and cultural values. For such people, wild animals constitute a valuable food resource, which cannot be easily withdrawn or replaced without causing wide-ranging socio-economic imbalances.

There is strong empirical evidence for the view that wildlife is being depleted on an unprecedented scale^3^ with a major transition in the scale of offtake in recent years. This drawdown is perceived by some as likely to have negative consequences for future generations^3,4^. Yet, conservation practitioners and planners often perceive hunting of wild animals as a drain to ecosystems^5,6^, in contrast to those involved with development issues who give greater emphasis to biodiversity as a resource to support human needs. Thus, to date, bushmeat has rarely figured seriously in international development strategies^3^, but has been a strong banner for the conservation lobby^7,8^. One reason for this may be that a strong relationship between use of wild meat and human health has not yet been fully confirmed.

Investigations of the role of wildlife on human health in central Africa are limited, most often restricted to isolated studies^2^ or based on estimated country-level production data from the Food Balance Sheets^4^ produced by the Food and Agriculture Organization of the United Nations (FAO). However, there is some evidence that indicates a strong causal link between bushmeat supply and human nutrition. For example, a study of children under 12 y of age in rural northeastern Madagascar showed that lack of access to wild meat causes a 29% increase in the numbers of children suffering from iron deficiency anemia and a tripling of anemia cases among children in the poorest households^9^. Thus, if consumption of sufficient amounts of nutrients to meet the body's needs are limited, including those contained in meats, chronic malnutrition will occur over time and will result in growth retardation in children (stunting) and eventually ill health in later life^10^.

In the absence of direct measures of nutritional status of human populations at a subnational level, stunting prevalence to the lowest administrative unit can be employed as a useful indicator of chronic malnutrition in Africa^11^. Stunting can then be used to correlate with the availability of different food items e.g. meats, even though various factors may affect retention of nutrients (e.g. disease^12,13,14^). Notwithstanding, in this paper we studied whether potential availability of wild meats was linked to stunting in children in central Africa. We base our analyses on the backdrop of the distribution of mammalian species assemblages, which we classify according to their hunting potential and in which we estimate wild meat biomass likely to be at the disposal of humans. Given the strong associations that appear between mammalian diversity areas and stunting, we then statistically test three plausible hypotheses to examine the association between stunting and huntable mammalian diversity as proxies of wild meat availability:

H_1_: Mammalian diversity patterns directly influence malnutrition in humans.

H_2_:Mammalian diversity patterns influence human population levels and their impacts and these are correlated with malnutrition in humans.

H_3_:Human population levels and their impacts influence both mammalian diversity areas and malnutrition in humans.

H_0_:There is no relationship between mammalian diversity patterns and human malnutrition.

We contend that if a strong correlation between bushmeat availability and malnutrition in humans is established, coalescing strategies that deal with conservation of wildlife, as well as human livelihoods, becomes imperative.

## Geographical focus

**Figure 1:**
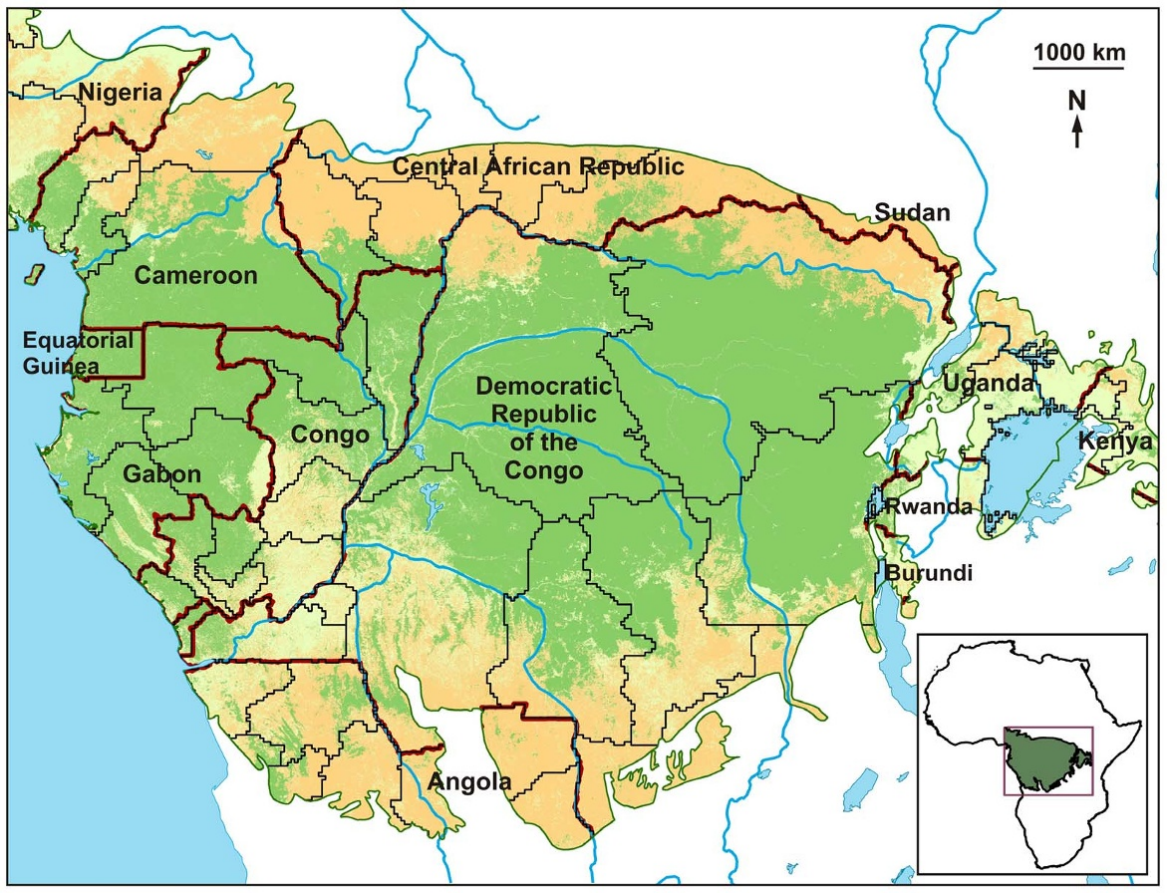
Study area. Green areas are rainforests and warm pink areas are woody savannas, taken from the Collection 5 MODIS Global Land Cover Type product (www.landcover.org). Coarse red lines are country borders and slim black lines are limits of subnational units considered by FAO for data on children stunting^20^. Maps were generated using ArcGIS.

Our study area was limited to the Rainforest Biotic Zone (RBZ) of central Africa. The RBZ, defined by Kingdon et al. (2013)^15^, following White (1983)^16^ encompasses six main countries (the Democratic Republic of the Congo, the Republic of the Congo, Central African Republic, Cameroon, Gabon and Equatorial Guinea), as well as parts of another three (Angola, Burundi and Rwanda) (Fig. 1). The main vegetation type in the region is Guineo-Congolian lowland rain forest, concentrated in the Congo basin, corresponding to the second largest (close to 2 million km^2^) and the least degraded area of contiguous moist tropical forest in the world^17^. Away from the central regions of the RBZ, the dominant vegetation includes woody savannas, as well as areas of cropland-natural vegetation mosaic^18^.

## Datasets

### Huntable mammal species

From a previous study^19^ in which we derived predicted distribution maps for all hunted terrestrial mammal species occurring within the RBZ, we delimited mammalian diversity areas for species of a lesser or greater hunting potential (see Methods). A total of 141 monotypic species and 24 others, including 67 subspecies, belonging to 11 Orders, were included in our analyses (see Methods and Appendix S1).

## Child stunting

**Figure 2:**
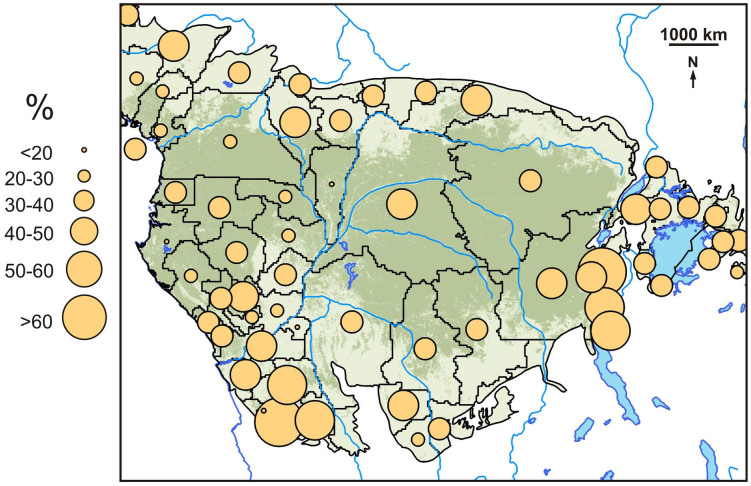
Prevalence of stunting among children under five^20^. Circles are located at the centroids of subnational units providing data. Circle size indicates prevalence. Map was generated using ArcGIS.

We used a global map of the distribution of chronic undernutrition at national and subnational levels depicting stunting in growth among children under five years of age^20^ (Fig. 2). This map, generated by the FAO, employs stunting as a measure of prevalence of chronic undernutrition. Stunting here is defined as height-for-age below minus two standard deviations from the international growth reference standard (National Center for Health Statistics/World Health Organization). This indicator reflects long-term cumulative effects of inadequate food intake and poor health conditions as a result of lack of hygiene and recurrent illness in poor and unhealthy environments.

## Results

### Huntable mammalian diversity and standing crop biomass

**Figure 3:**
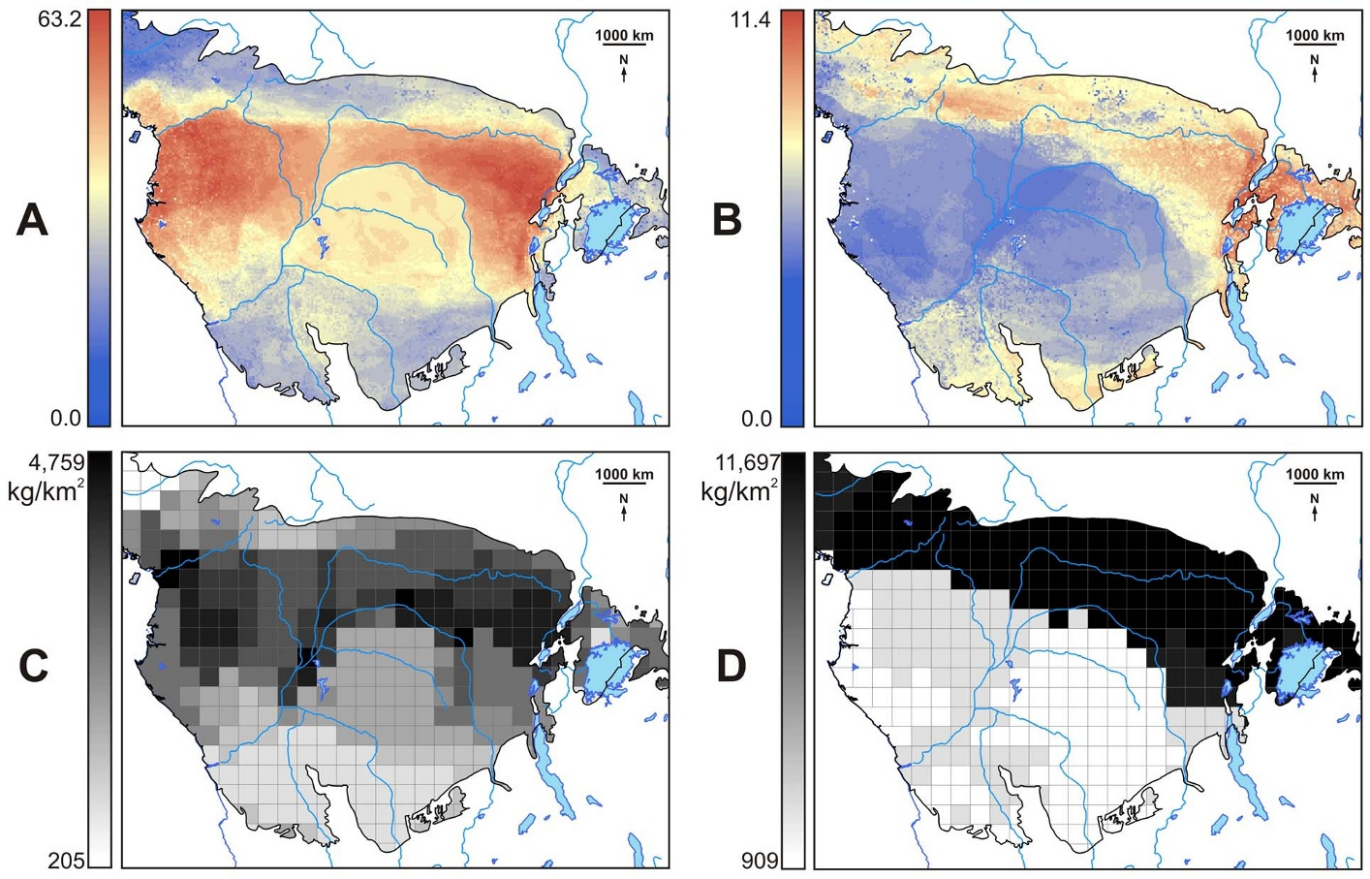
Diversity and standing biomass of mammals in central Africa. (A) Deep Rainforest Diversity, DRD. (B) Marginal Rainforest Diversity, MRD. DRD and MRD are the accumulated favorability values, weighted by hunting sustainability values, of all hunted mammals (Appendix S1) found within the Rainforest Biotic Zone. (C) Potential standing biomass in DRD mammals. (D) Potential standing biomass in MRD mammals. Maps were generated using ArcGIS.

We distinguished two separate mammalian assemblages: (1) a Deep Rainforest Diversity (DRD), largely composed of low hunting-resilient species i.e. large-bodied, slow reproducing taxa, mostly found within wet Guinea-Congolian lowland rainforest in the center of the RBZ (Fig. 3A) and (2) a Marginal Rainforest Diversity (MRD), comprised of high hunting-resilient taxa, i.e. smaller-bodied, fast-reproducing mammals inhabiting the woody savanna/grasslands in the northern, eastern and southern RBZ^21^ (Fig. 3B).

Total standing crop mammalian biomass within each mammalian assemblage correlated significantly and positively with both DRD (*n* = 367 grid cells; *r* = 0.167; *P* < 0.001) and MRD areas (*n* = 367 grid cells; *r* = 0.595; *P* < 0.001). However, potential standing biomass of mammal species of low hunting potential^19^ was significantly and positively correlated with DRD areas (*n* = 367 grid cells; *r* = 0.652; *P* < 0.001). Likewise, the potential standing biomass of mammal species of high hunting potential^19^ was significantly and positively correlated with MRD areas (*n* = 367 grid cells; *r* = 0.773; *P* < 0.001).

Using standing biomass as a surrogate of potential wild meat resources available to humans, we showed that higher mammalian biomass was typical of MRD but not of DRD areas, despite the latter areas having six times more diversity than MRD areas (Figs. 3A and 3B). Potential standing biomass in DRD areas (Fig. 3C) was lower (mean ± SE = 1,805 ± 1,074 kg/km^2^, median = 1,535 kg/km^2^, range = 205–4,759 kg/km^2^) than that in MRD areas (Fig. 3D) (mean ± SE = 5,618 ± 4,296 kg/km^2^, median = 2,461 kg/km^2^, range = 909–11,697 kg/km^2^).

### Bushmeat extraction patterns

**Figure 4:**
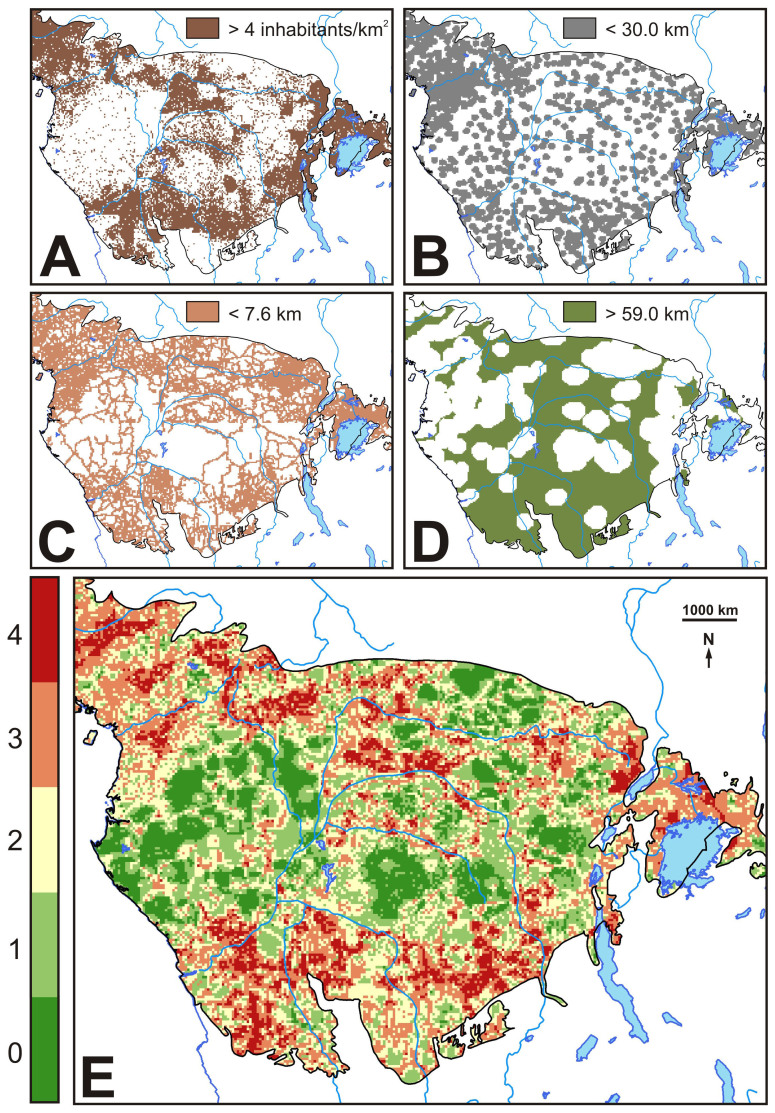
Anthropogenic pressures. (A) Brown: above median areas of rural human population density. (B) Grey: below median areas of distance to urban areas. (C) Pink: below median areas of distance to roads. (D) Green: above median areas of distance to protected areas. (E) Bushmeat extraction patterns emerging from the overlay of urban areas, road networks, protected areas and densely populated rural areas (areas with a total score of 4 had the highest bushmeat extraction potential, whereas areas with a total score of a 0 had the lowest). Maps were generated using ArcGIS.

The overlay map of urban, road networks, protected areas and densely populated rural areas within the RBZ (see Methods) (Fig. 4) indicated that potential hunting intensity was higher in the MRD areas but lower in the DRD areas.

### Stunting, mammalian diversity and standing biomass

Stunting was unevenly distributed throughout the study region with more stunting occurring away from the central DRD areas (Fig. 2). Stunting was negatively correlated with mammalian diversity in DRD areas (*n* = 60; *r* = −0.288; *P* = 0.027) but positively associated to MRD areas (*n* = 60; *r* = 0.325; *P* = 0.012). Bushmeat extraction values were positively correlated with the prevalence of child stunting (*n* = 60; *r* = 0.373; *P* < 0.005) and with mammalian diversity in the MRD areas (*n* = 60; *r* = 0.484; *P* < 0.001). Extraction was negatively correlated with mammalian diversity in the DRD areas (*n* = 60; *r* = −0.469; *P* < 0.001).

### Hypothesis testing

**Figure 5:**
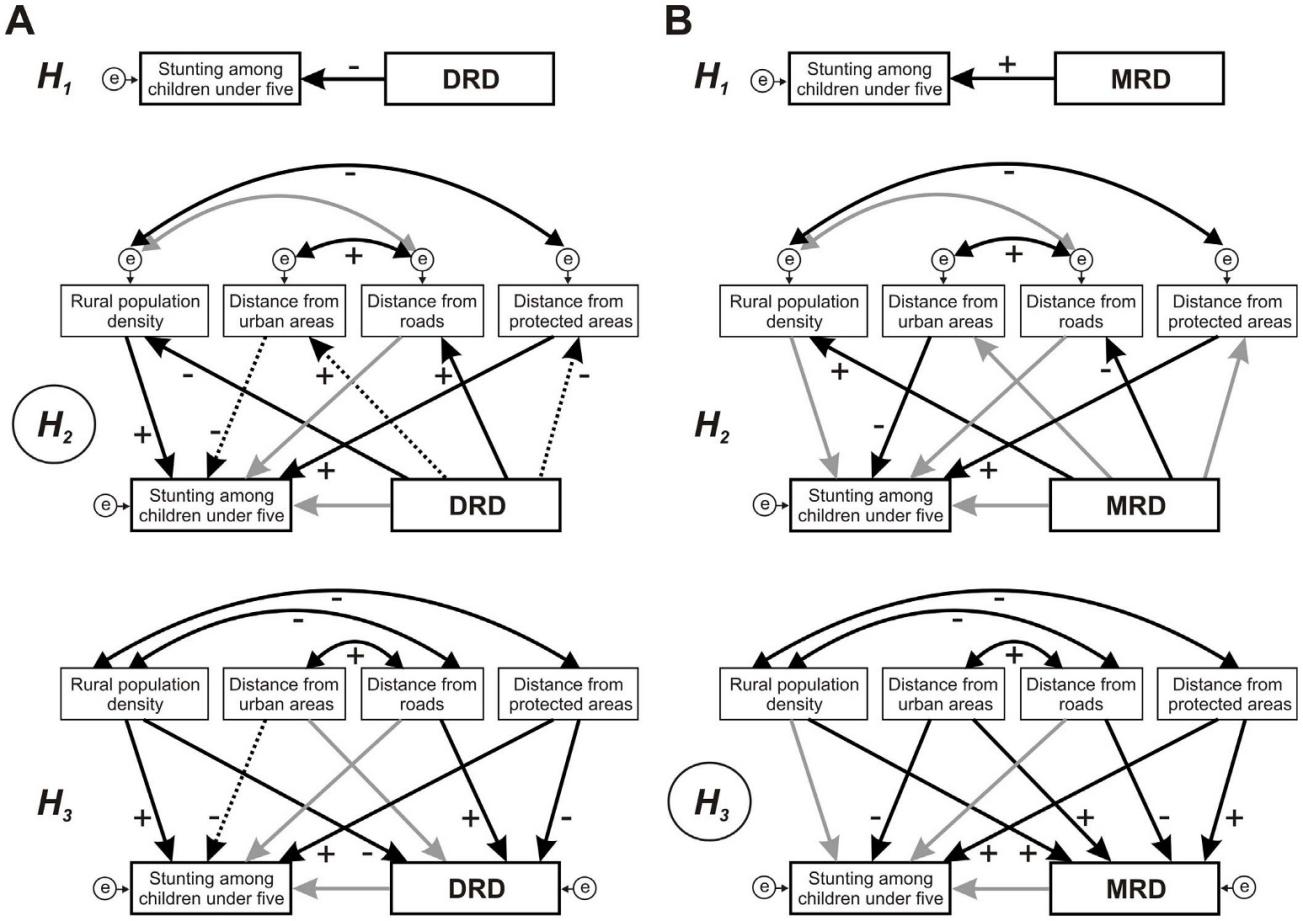
Path diagrams representing relationships between diversity and stunting among children. Three hypotheses are tested: ***H***_**1**_ (direct relationship); ***H***_**2**_ (diversity influences human variables and these influence stunting); ***H***_**3**_ (human variables influence both diversity and stunting). (A) Models for Deep Rainforest Diversity (DRD). (B) Models for Marginal Rainforest Diversity (MRD). Circles enclosing "e": error terms associated to dependent variables. Solid black arrows: significant relationships (n = 60; *P* < 0.05); dashed black arrows: 0.05 < *P* < 0.07; grey arrows: non-significant relationships (P > 0.07); double arrows: covariance between variables, which are considered in the diagrams when significant correlations were identified within the study area (*P* < 0.05). +: Positive relationship; - : Negative relationship. Encircled hypotheses (*H*_2_ in **A** and *H*_3_ **B**) indicate the best fitted models.

We found no evidence to support H_1_ for both DRD and MRD areas. Any direct relationship lost statistical significance when other factors were included in the models (Fig. 5; Table 1). Domestic meat was excluded from all models because it neither showed significant relationships with stunting among children nor influenced the rest of relationships among variables (compare Table 1 and Fig. 5 with Table S1 and Fig. S1 in Appendix S2). The inclusion of domestic meats enlarged the differences between observed and expected covariance matrices (see χ^2^ in Table S2, Appendix S2).

**Table 1:** Standardized weights (SW) and statistical significance (P) of regressions. Hypotheses tested for the relationship between mammal diversity and stunting: H_1_ (direct relationship); H_2_ (diversity influences human variables and these influence stunting); H_3_ (human variables influence both diversity and stunting). DRD: Deep Rainforest Diversity; MRD: Marginal Rainforest Diversity. To identify dependent and independent variables, see Fig. 5

	Diversity		Stunting prevalence	
	SW	*P*	SW	*P*
H_1_ **- DRD**				
**DRD**			−0.288	0.022
H_2_ **-DRD**				
**DRD**			−0.024	0.869
**Rural population density**	−0.394	0.001	0.342	0.016
**Distance from urban areas**	0.235	0.066	−0.264	0.058
**Distance from roads**	0.509	<0.001	0.012	0.939
**Distance from protected areas**	−0.234	0.067	0.323	0.014
H_3_ **- DRD**				
**DRD**			−0.024	0.870
**Rural population density**	−0.358	0.003	0.351	0.015
**Distance from urban areas**	−0.098	0.427	−0.268	0.054
**Distance from roads**	0.391	0.003	0.012	0.940
**Distance from protected areas**	−0.364	<0.001	0.328	0.015
H_1_ **- MRD**				
**MRD**			0.325	0.009
H_2_ **- MRD**				
**MRD**			0.145	0.376
**Rural population density**	0.643	<0.001	0.261	0.117
**Distance from urban areas**	−0.103	0.432	−0.297	0.035
**Distance from roads**	−0.444	<0.001	0.044	0.781
**Distance from protected areas**	0.019	0.886	0.298	0.019
H_3_ **- MRD**				
**MRD**			0.149	0.378
**Rural population density**	0.643	<0.001	0.263	0.125
**Distance from urban areas**	0.231	0.030	−0.300	0.036
**Distance from roads**	−0.269	0.018	0.043	0.781
**Distance from protected areas**	0.241	0.011	0.301	0.020

H_2_ was more consistent than H_3_ for DRD areas, (Fig. 5A; Table 2). Arrow signs linking DRD areas with the four main human variables were opposite to those linking these variables to bushmeat extraction. This suggests that remoteness to human agglomerations and infrastructures was linked to lower levels of bushmeat offtake. Moreover, child stunting was higher in rural and urban areas of higher human concentrations, but lower in those areas closer to protected areas.

**Table 2:** Fit summary for models relating mammal diversity, stunting and human pressure. χ^2^: test of differences between observed and expected covariance matrices; *P*: statistical significance of χ^2^; TLI: Tucker-Lewis Index; CFI: Comparative Fit Index; NFI: Normed Fit Index; RMSEA: Root Mean Square Error of Approximation; AIC: Akaike Information Criterion. Hypotheses tested for the relationship between diversity and stunting: H_2_ (diversity influences human variables and these influence stunting); H_3_ (human variables influence both diversity and stunting). DRD: Deep Rainforest Diversity; MRD: Marginal Rainforest Diversity. All statistics show best fit of H_2_ for DRD and of H_3_ for MRD

	χ^2^	*P*_[d.f. = 3] = 3]_	TLI	CFI	NFI	RMSEA	AIC
H_2_ **- DRD**	3.572	0.312	0.964	0.993	0.962	0.057	51.572
H_3_ **- DRD**	4.573	0.206	0.900	0.980	0.951	0.095	52.573
H_2_ **- MRD**	4.905	0.179	0.898	0.980	0.955	0.105	52.905.
H_3_ **- MRD**	4.573	0.206	0.916	0.983	0.958	0.095	52.573

In MRD areas, H_3_ was better supported than H_2_ (Fig. 5B; Table 2). In this model, arrow signs linking the human variables with MRD were the same as those linking human variables to bushmeat extraction, with the exception of a positive relationship with distance to urban areas.

## Discussion

There is growing evidence that forest cover and dietary diversity are correlated in Africa^22^. Forest foods help maintain household nutrition in many communities, especially during lean seasons (complementing, for example, the seasonality of staple agricultural crops), in times of low agricultural production, during periods of climate-induced vulnerability and when gaps in the availability of food occur due to other cyclical events. Animal source food consumption, however, was not related to tree cover^23^, perhaps because wildlife is the main source of nutrients in many tropical forest and non-forest regions^2,24^. A significant proportion of the wildlife biomass hunted by humans for food across the tropics, especially large-bodied primates, ungulates and rodents (average weight greater than 1 kg), is found in tropical rainforests, with ungulates and sometimes rodents dominating the biomass in more open habitats^25^. Animal-based foods supply many important micronutrients in much higher amounts or with higher bioavailability than most plant-based foods^26^ and as attested in Golden et al.'s (2011)^9^ study on the importance of wild meat in reducing iron deficiency anemia in children.

There are growing concerns that any decline in the availability of wild meat will threaten the food security and livelihoods of forest communities^27^, especially those in which home consumption is more common than wild-meat trading. However, the relationship between wild meat availability and human nutrition may vary according to habitat type and region. In our study, we show, for the first time, that the relationship between hunted mammalian diversity, which in turn is linked to wild meat availability and human pressure, are correlated with children malnutrition levels. We show that the more remote forest areas within central African rainforests seem capable of adequately supporting existing human populations at a reasonable level of health. This contrasts with the more highly populated woody savannas/grasslands along the northern, eastern and southern RBZ that are under much higher anthropogenic pressures i.e. more bushmeat extraction. Hence, this spatial disparity in human needs and bushmeat supply present challenges for development and conservation.

Although our analyses are based on inferences made from correlations between interacting environmental and nutrition variables, our results correspond with others on the state of conservation of habitats and fauna in the Congo Basin. For example, our data points to the importance of the central rainforest blocks as significant regions of continued forest protection^28^. Such ‘deep forest’ faunas, though also under substantial threats^28^, are currently under less anthropogenic pressures than the ecotonal regions along the margins of the RBZ. Human activity in these more open habitats, primarily burning and land clearing for cultivation is intense^29^. Moreover, anthropogenic disturbance around cities has led to significant decreases in faunal diversity^30^. In our MRD model, proximity to urban areas is the only human-pressure variable significantly explaining stunting, but closer to protected areas stunting is less.

An immediate consequence of our study should be to rouse producer governments to put appropriate management regimes in place to integrate the bushmeat issue into the discussion on assessment of environmental assets. This is not new^7,8^, but here we advance the debate by presenting a more complex scenario, in which deep rainforest wildlife may still support food security of hunter-gatherers and others on condition that human concentrations are kept low. Instead, along the RBZ margins, composed of more sustainable wildlife and more productive in terms of wild meat, higher population densities here explain the observed levels of malnutrition. More specifically, adequate human nutrition is likely in rural landscapes, but as our analyses show, collapses around urban areas, where child malnutrition is more prevalent. Although our results require further empirical tests and more work on the ground to investigate how the different drivers affect malnutrition and the role wildlife plays, the strong correlations we confirm between wild meat and malnutrition are noteworthy. We thus argue that this is not a spurious effect, but one that powerfully points to the significance of wild meat in sustaining human populations in central Africa. However, our results should be considered of heuristic value and this stage not to be used to propose unfettered access rights for the poor nor draconian conservation schemes. What it does underline, rather, is the need to consider a wider political agenda for developing practical policies that benefit both people and biodiversity. Emerging strategies from this framework would increase public recognition of bushmeat's economic value and the need to regulate and plan its use, but it would also emphasize the need for adequate and accessible alternative food sources to overturn the malnutrition levels seen along the marginal RBZ habitats. All this would raise an interesting set of questions about (for example) the relationship between natural resource use and economic growth, or between effective conservation and resilient development.

## Methods

### Mapping mammalian assemblages

We distinguished two separate mammalian assemblages, DRD and MRD, in central Africa based on Fa et al.'s (2014)^19^ analysis of differing capacity for hunting sustainability of each species. For this, we employed two indices based on accumulating favorability values obtained through distribution modeling, for every species in every locality^19,31^. Favorability models define to which degree environmental conditions at each locality favor the species' presence, independently of the species' prevalence^32,33^. We built favorability models for 165 species (see Datasets section and Appendix S1) using 1° × 1°-resolution presences and absences derived from IUCN's (2014)^34^ range maps. At this spatial resolution, models based on extent-of-occurrence maps are still meaningful^35^. We trained the models using 27 variables describing climate, topo-hydrography, land cover/use and other anthropogenic forces (see Fa et al. 2014^19^ for more details). We then used the "direct downscaling approach" to project all models to a 0.1° × 0.1° resolution grid^36^. Only favorability values where species are known to occur according to the IUCN (2014)^34^ were retained. Here, favorability values for every subspecies were considered separately.

MRD values corresponded to the "Sustainable Accumulated Favorability" (SAF_j_) in Fa et al. (2014)^19^. This index was calculated by adding up the favorability value (F_i_) of all i taxa in each j cell in the study area, after each taxon's favorability was weighted according to the taxon's potential resilience to hunting (Potential Hunting Sustainability, PHS, see the "restrictive" approach in Fa et al. 2014^19^). PHS was measured according to four ecological traits that are linked with extinction proneness^19,37^: population density, habitat breadth, rarity and vulnerability. SAF_j_ (and so MRD) was finally computed as follows:DRD values corresponded, instead, to the "Unustainable Accumulated Favorability" (UAF_j_) in Fa et al. (2014)^19^, which was computed as follows:Mapping SAF_j_ and UAF_j_ revealed the existence of two partially disjoint mammalian assemblages, respectively located in the northern, eastern and southern margins of the rainforest region (hence MRD) and in the Guinea-Congolian rainforest blocks (hence DRD).

### Mammalian standing biomass

We assessed wild meat availability by estimating the standing crop mammalian biomass existing in a 1° × 1°-resolution grid of the study region. Standing biomass was estimated as a function of the number of occurring species (>1 kg in weight and known to be hunted^19^), the mean population density of every species and the mean body size of each species' individuals. Species occurrences were taken from IUCN (2014)^34^, body sizes from Kingdon et al. (2013)^15^ and population densities derived from various sources. Mean density data for 53 (32%) species were taken from the PanTHERIA world mammal database^38^; 15 (9%) from Fa & Purvis (1997)^39^; and for 97 (59%) other taxa we derived expected values from the linear regression of log population density on log body mass. This regression, of high statistical significance (*n* = 949 species; *r* = 0.574; *P* < 0.001), was performed using data contained in PanTHERIA^38^.

To calculate the potential mammal standing biomass of a given 1° × 1° grid cell, we first multiplied, for every species occurring in the grid, its mean population density and mean body size. We then summed the products of these multiplications. Four species [savanna elephant (*Loxodonta africana*), forest elephant (*L. cyclotis*), hippopotamus (*Hippopotamus* amphibius) and forest buffalo (*Syncerus caffer nanus*)] were excluded from our calculations because, although hunted for meat^40^, are only occasional prey and thus do not represent an important source of wild meat.

Potential standing biomass in mammals of low hunting potential was calculated considering only species with Potential Hunting Sustainability (PHS) < (mean PHS - standard error, SE) (i.e. PHS < 0.06 in Appendix S1). Likewise, to calculate the potential standing biomass of mammal species of high hunting potential we considered all species with PHS > (mean PHS + SE) (i.e. PHS > 0.09 in Appendix S1).

### Bushmeat extraction patterns

The concentration of human populations, their accessibility to hunting areas, as well as the presence of protected areas have been reported as significant predictors of bushmeat extraction intensity in the Congo Basin^41^. From this, we considered four relevant anthropogenic variables in our models that could determine potential bushmeat extraction levels in our study area: (1) rural human population density— assumed to be the population fraction engaged in hunting^42^—, (2) proximity to urban areas—representing non-subsistence bushmeat demanding areas^43^—, (3) proximity to roads— as a measure of access to hunting areas—and (4) distance to protected areas — often reservoirs areas for many species (for variable sources, see Supplementary Information). We estimated the spatial distribution of potential bushmeat extraction throughout the RBZ, by first classifying each of the four variables in 0.1° × 0.1° resolution maps with a 1 if above the median and with a 0 if below the median. Resulting maps for each variable were finally summed, so that areas with a total score of 4 had the highest bushmeat extraction potential, whereas areas in with a total score of a 0 had the lowest. We assessed the suitability of our proxy by testing the correlation with Ziegler et al.'s^41^ (in press) model in the Congo Basin, using average values for both estimations on 1° × 1° grids. Our extraction model and Ziegler's et al. were highly correlated (*n* = 60; *r* = 0.803; *P* < 0.001).

### Statistical methods

The consistence of the above-listed hypotheses was tested using Structural Equation Modelling^44^. A set of interrelated variables were linked to each-other according to a priori models following the working hypotheses (Appendices 3 and 4), which were designed as diagrams describing a system of possible relationships among response and predictor variables (Fig. 5 and Fig. S1 in Appendix S1). These variables were DRD, MRD, prevalence of stunting among children, rural human population density, distance to urban areas, distance to roads, distance to protected areas and domestic meat (for variable sources, see Supplementary Information). Structural Equation Modelling, basically an extension of Path Analysis^45^ allowing for model comparison, was used to assess the diagrams (hypothesis-testing studies using this approach^46,47,48^). Cause-and-effect relationships were depicted by one-headed arrows and every arrow was given a path coefficient that can be either significant or not. This coefficient is a standard partial regression coefficient^45^ and measures the strength of a relationship as a proportion of the total standard deviation (Table 1). Thus, variables that, in isolation, are highly correlated can be given low path coefficients as a result of indirect relationships between third variables. Covariances between independent variables were considered in the diagrams when significant correlations were identified within the study area (*n* = 60; *P* < 0.05). We used 60 sub-national administrative units as the basis for the analysis (Fig. 1), because the original data of stunting among children were only available on this geographical support^20^. We, thus, used average values of the rest of variables, referred to the 60 units of reference.

The goodness of fit of each structural equation model to data was assessed using five parameters (table S2): (1) a χ^2^ statistic test of the differences between observed and expected covariance matrices, quantified by a likelihood function^49^; (2) the Tucker-Lewis Index (TLI)^50^; (3) the Comparative Fit Index (CFI)^50^; (4) the Normed Fit Index (NFI)^51,52^; (5) the Root Mean Square Error of Approximation (RMSEA)^53,54^; the Akaike Information Criterion (AIC)^55^. Accepting a model requires χ^2^ being non-significant and as small as possible; TLI, CFI and NFI values close to one indicate a very good fit; RMSEA should be lower than 0.1 and as small as possible. The best model should minimize AIC as well.

## Electronic supplementary material

Supplementary Information
Supplementary Information
