# Supplementary material for: Disentangling the relative effects of bushmeat availability on human nutrition in central Africa
Source: Sci Rep. 2015 Feb 2;5:8168. doi: 10.1038/srep08168 (PMC4313087; doi:10.1038/srep08168)
Supplement: Supplementary file 1 — Supplementary Information Supplementary Information [file 41598_2015_BFsrep08168_MOESM1_ESM.doc]

Supplementary Material

Disentangling the relative effects of bushmeat availability on human nutrition in central Africa

Julia E. Fa^1,2,7^, Jesús Olivero^2^, Raimundo Real^2^, Miguel A. Farfán^2^, Ana L. Márquez^2^, J. Mario Vargas^2^, Stefan Ziegler^3^, Martin Wegmann^4^, David Brown^5^, Barrie Margetts^6^ & Robert Nasi^7^

1 ICCS, Division of Biology, Imperial College London, Ascot SL5 7PY, UK;

2 Grupo de Biogeografía, Diversidad y Conservación, Universidad de Málaga, 29071 Málaga, Spain;

3 WWF Germany, 10117 Berlin, Germany and Department of Ecology and Evolution, University Frankfurt, 60438 Frankfurt on Main, Germany;

4 DLR Berlin, German Aerospace Center (DLR), 82234 Wessling, Germany, and Department of Remote Sensing, Department of Geography and Geology, University of Würzburg, 97074 Würzburg, Germany;

5 Overseas Development Institute, London SE1 8NJ, UK;

6 Faculty of Medicine, University of Southampton, Southampton SO16 6YD, UK;

7 Consultative Group on International Agricultural Research (CGIAR), CIFOR Headquarters, Bogor 16115, Indonesia.

**Appendix S1:** Mammalian diversity areas.

**Table S1: List of mammal taxa used to build indices for Deep Rainforest Diversity (DRD) and Marginal Rainforest Diversity (MRD).** The distinction between these types of faunas arose when species and subspecies were weighted according to their potential hunting sustainability (**PHS**)*. All taxa are listed below according to **PHS**. **CS**: sustainability category* (sustainability increases from 1 to 5). **ABW**: adult body weight (g)**.

| **Order** | | **Species** | **PHS** | **CS** | **ABW** |
| --- | --- | --- | --- | --- | --- |
| *Hyracoidea* | *Procavia capensis* | | 1.0000 | 5 | 2952.5 |
| *Carnivora* | *Herpestes sanguineus* | | 0.8352 | 5 | 507.5 |
| *Carnivora* | *Ictonyx striatus* | | 0.7062 | 5 | 811.0 |
| *Carnivora* | *Mellivora capensis* | | 0.6753 | 5 | 9000.0 |
| *Carnivora* | *Genetta maculata* | | 0.4973 | 5 | 1950.0 |
| *Primates* | *Galago senegalensis* | | 0.4353 | 5 | 215.2 |
| *Rodentia* | *Xerus erythropus* | | 0.4279 | 5 | 602.2 |
| *Carnivora* | *Caracal caracal* | | 0.3888 | 5 | 11964.4 |
| *Carnivora* | *Herpestes ichneumon* | | 0.3862 | 5 | 2980.0 |
| *Carnivora* | *Helogale parvula* | | 0.3804 | 5 | 281.8 |
| *Carnivora* | *Hydrictis maculicollis* | | 0.3187 | 5 | 5000.0 |
| *Rodentia* | *Cricetomys gambianus* | | 0.3093 | 5 | 1267.5 |
| *Carnivora* | *Poecilogale albinucha* | | 0.3071 | 5 | 308.2 |
| *Carnivora* | *Leptailurus serval* | | 0.3027 | 5 | 12000.0 |
| *Primates* | *Galagoides demidovii* | | 0.2975 | 5 | 64.0 |
| *Primates* | *Galagoides thomasi* | | 0.2918 | 5 | 89.0 |
| *Artiodactyla* | *Philantomba monticola* | | 0.2649 | 5 | 4896.1 |
| *Rodentia* | *Heliosciurus gambianus* | | 0.2643 | 5 | 248.4 |
| *Rodentia* | *Anomalurus derbianus* | | 0.2609 | 5 | 665.8 |
| *Primates* | *Otolemur crassicaudatus* | | 0.2529 | 5 | 1206.6 |
| *Primates* | *Galago moholi* | | 0.2519 | 5 | 192.0 |
| *Rodentia* | *Thryonomys swinderianus* | | 0.2512 | 5 | 3750.9 |
| *Tubulidentata* | *Orycteropus afer* | | 0.2491 | 5 | 56175.2 |
| *Carnivora* | *Mungos mungo* | | 0.2434 | 5 | 1260.0 |
| *Primates* | *Papio anubis* | | 0.2224 | 5 | 17728.6 |
| *Pholidota* | *Phataginus tricuspis* | | 0.2177 | 5 | 2480.0 |
| *Rodentia* | *Cricetomys emini* | | 0.2066 | 5 | 1280.0 |
| *Carnivora* | *Ichneumia albicauda* | | 0.1955 | 5 | 3628.4 |
| *Afrosoricida* | *Potamogale velox* | | 0.1743 | 5 | 671.0 |
| *Rodentia* | *Hystrix africaeaustralis* | | 0.1718 | 5 | 14936.0 |
| *Carnivora* | *Civettictis civetta* | | 0.1687 | 5 | 12075.6 |
| *Carnivora* | *Genetta genetta* | | 0.1660 | 5 | 1756.2 |
| *Rodentia* | *Atherurus africanus* | | 0.1642 | 4 | 2875.7 |
| *Rodentia* | *Anomalurus beecrofti* | | 0.1638 | 4 | 479.1 |
| *Rodentia* | *Protoxerus stangeri* | | 0.1632 | 4 | 630.4 |
| *Rodentia* | *Heliosciurus rufobrachium* | | 0.1573 | 4 | 332.9 |
| *Rodentia* | *Paraxerus cepapi* | | 0.1534 | 4 | 222.9 |
| *Primates* | *Chlorocebus pygerythrus* | | 0.1487 | 4 | 4150.0 |
| *Primates* | *Chlorocebus tantalus* | | 0.1424 | 4 | 2800.0 |
| *Rodentia* | *Funisciurus congicus* | | 0.1380 | 4 | 112.0 |
| *Rodentia* | *Funisciurus anerythrus* | | 0.1299 | 4 | 223.5 |
| *Artiodactyla* | *Tragelaphus scriptus* | | 0.1283 | 4 | 43250.4 |
| *Artiodactyla* | *Phacochoerus africanus* | | 0.1251 | 4 | 82500.0 |
| *Carnivora* | *Nandinia binotata* | | 0.1241 | 4 | 2167.2 |
| *Rodentia* | *Hystrix cristata* | | 0.1224 | 4 | 13406.3 |
| *Rodentia* | *Funisciurus pyrropus* | | 0.1211 | 4 | 243.2 |
| *Primates* | *Colobus guereza occidentalis* | | 0.1084 | 4 | 9925.9 |
| *Carnivora* | *Genetta thierryi* | | 0.1071 | 4 | 1400.0 |
| *Hyracoidea* | *Dendrohyrax dorsalis* | | 0.1068 | 4 | 3175.0 |
| *Rodentia* | *Paraxerus poensis* | | 0.1003 | 4 | 100.0 |
| *Primates* | *Chlorocebus cynosuros* | | 0.0979 | 4 | 4550.0 |
| *Pholidota* | *Phataginus tetradactyla* | | 0.0969 | 4 | 2785.0 |
| *Primates* | *Papio cynocephalus* | | 0.0951 | 4 | 15822.2 |
| *Carnivora* | *Xenogale naso* | | 0.0941 | 4 | 2900.0 |
| *Primates* | *Cercopithecus neglectus* | | 0.0897 | 3 | 5324.5 |
| *Hyracoidea* | *Dendrohyrax arboreus* | | 0.0891 | 3 | 2981.1 |
| *Artiodactyla* | *Syncerus caffer caffer* | | 0.0815 | 3 | 592666.0 |
| *Rodentia* | *Paraxerus boehmi* | | 0.0639 | 2 | 70.2 |
| *Primates* | *Perodicticus potto edwardsi* | | 0.0616 | 2 | 1081.8 |
| *Proboscidea* | *Loxodonta africana* | | 0.0615 | 2 | 3824539.9 |
| *Primates* | *Cercopithecus ascanius schmidti* | | 0.0613 | 2 | 3540.2 |
| *Artiodactyla* | *Hyemoschus aquaticus* | | 0.0611 | 2 | 10850.0 |
| *Carnivora* | *Bdeogale crassicauda* | | 0.0577 | 2 | 1722.1 |
| *Carnivora* | *Crossarchus alexandri* | | 0.0577 | 2 | 1503.4 |
| *Pholidota* | *Smutsia gigantea* | | 0.0561 | 2 | 32500.0 |
| *Artiodactyla* | *Syncerus caffer nanus* | | 0.0552 | 2 | 592666.0 |
| *Artiodactyla* | *Cephalophus dorsalis* | | 0.0536 | 2 | 20000.0 |
| *Carnivora* | *Atilax paludinosus* | | 0.0528 | 2 | 3600.2 |
| *Carnivora* | *Felis silvestris* | | 0.0518 | 2 | 4573.1 |
| *Carnivora* | *Aonyx congicus* | | 0.0511 | 2 | 24000.0 |
| *Artiodactyla* | *Tragelaphus spekii* | | 0.0495 | 2 | 75554.3 |
| *Artiodactyla* | *Cephalophus rufilatus* | | 0.0482 | 2 | 12114.6 |
| *Primates* | *Colobus angolensis angolensis* | | 0.0447 | 2 | 8990.3 |
| *Artiodactyla* | *Hippotragus equinus* | | 0.0432 | 2 | 264174.0 |
| *Artiodactyla* | *Cephalophus nigrifrons* | | 0.0404 | 2 | 14676.3 |
| *Artiodactyla* | *Hippopotamus amphibius* | | 0.0383 | 2 | 1536310.4 |
| *Carnivora* | *Aonyx capensis* | | 0.0379 | 2 | 19322.2 |
| *Primates* | *Arctocebus aureus* | | 0.0378 | 2 | 234.2 |
| *Rodentia* | *Funisciurus lemniscatus* | | 0.0372 | 2 | 141.0 |
| *Rodentia* | *Funisciurus isabella* | | 0.0360 | 2 | 109.3 |
| *Primates* | *Cercopithecus nictitans nictitans* | | 0.0351 | 2 | 5256.9 |
| *Artiodactyla* | *Tragelaphus oryx* | | 0.0319 | 2 | 419000.0 |
| *Artiodactyla* | *Cephalophus weynsi* | | 0.0319 | 2 | 17300.0 |
| *Primates* | *Cercopithecus ascanius katangae* | | 0.0318 | 2 | 3540.2 |
| *Primates* | *Cercocebus agilis* | | 0.0297 | 2 | 7105.6 |
| *Primates* | *Sciurocheirus gabonensis* | | 0.0296 | 2 | 260.0 |
| *Carnivora* | *Genetta servalina* | | 0.0288 | 2 | 1175.8 |
| *Artiodactyla* | *Cephalophus silvicultor* | | 0.0288 | 2 | 62006.6 |
| *Carnivora* | *Crossarchus ansorgei* | | 0.0285 | 2 | 700.0 |
| *Artiodactyla* | *Syncerus caffer aequinoctialis* | | 0.0284 | 2 | 592666.0 |
| *Primates* | *Cercopithecus cephus* | | 0.0277 | 2 | 3444.9 |
| *Rodentia* | *Myosciurus pumilio* | | 0.0275 | 2 | 16.4 |
| *Primates* | *Cercopithecus ascanius whitesidei* | | 0.0270 | 2 | 3540.2 |
| *Rodentia* | *Epixerus ebii* | | 0.0269 | 2 | 521.0 |
| *Primates* | *Cercopithecus mitis stuhlmanni* | | 0.0268 | 2 | 5041.3 |
| *Primates* | *Euoticus elegantulus* | | 0.0258 | 2 | 295.5 |
| *Carnivora* | *Dologale dybowskii* | | 0.0256 | 2 | 361.9 |
| *Primates* | *Lophocebus aterrimus* | | 0.0255 | 2 | 6510.4 |
| *Primates* | *Cercopithecus pogonias grayi* | | 0.0250 | 2 | 3578.3 |
| *Carnivora* | *Canis adustus* | | 0.0245 | 2 | 9658.7 |
| *Artiodactyla* | *Cephalophus callipygus* | | 0.0238 | 2 | 19079.2 |
| *Rodentia* | *Funisciurus bayonii* | | 0.0236 | 2 | 135.0 |
| *Artiodactyla* | *Cephalophus leucogaster* | | 0.0234 | 2 | 13208.7 |
| *Primates* | *Cercopithecus ascanius ascanius* | | 0.0232 | 2 | 3540.2 |
| *Primates* | *Perodicticus potto ibeanus* | | 0.0231 | 2 | 1081.8 |
| *Rodentia* | *Funisciurus leucogenys* | | 0.0231 | 2 | 250.0 |
| *Artiodactyla* | *Potamochoerus larvatus* | | 0.0228 | 2 | 69063.8 |
| *Primates* | *Lophocebus albigena* | | 0.0227 | 2 | 7418.7 |
| *Artiodactyla* | *Hylochoerus meinertzhageni* | | 0.0227 | 2 | 198130.5 |
| *Primates* | *Lophocebus johnstoni* | | 0.0227 | 2 | 7418.7 |
| *Carnivora* | *Crossarchus platycephalus* | | 0.0220 | 2 | 1108.0 |
| *Artiodactyla* | *Syncerus caffer brachyceros* | | 0.0218 | 2 | 592666.0 |
| *Primates* | *Miopithecus ogouensis* | | 0.0206 | 2 | 1255.0 |
| *Carnivora* | *Crocuta crocuta* | | 0.0189 | 2 | 63370.0 |
| *Primates* | *Cercopithecus mona* | | 0.0187 | 2 | 3980.2 |
| *Rodentia* | *Paraxerus alexandri* | | 0.0180 | 2 | 50.7 |
| *Primates* | *Procolobus rufomitratus oustaleti* | | 0.0178 | 2 | 8675.0 |
| *Primates* | *Cercopithecus wolfi wolfi* | | 0.0171 | 2 | 3255.2 |
| *Primates* | *Miopithecus talapoin* | | 0.0169 | 2 | 1248.9 |
| *Primates* | *Perodicticus potto potto* | | 0.0165 | 2 | 1081.8 |
| *Primates* | *Procolobus rufomitratus tholloni* | | 0.0163 | 2 | 8675.0 |
| *Carnivora* | *Poiana richardsonii* | | 0.0161 | 2 | 570.1 |
| *Primates* | *Allenopithecus nigroviridis* | | 0.0159 | 2 | 4750.0 |
| *Primates* | *Cercopithecus denti* | | 0.0138 | 2 | 3500.0 |
| *Primates* | *Cercopithecus nictitans martini* | | 0.0135 | 2 | 5256.9 |
| *Rodentia* | *Anomalurus pusillus* | | 0.0130 | 2 | 250.0 |
| *Primates* | *Colobus satanas anthracinus* | | 0.0125 | 2 | 9055.3 |
| *Primates* | *Cercocebus torquatus* | | 0.0123 | 2 | 7293.1 |
| *Artiodactyla* | *Potamochoerus porcus* | | 0.0119 | 2 | 70000.3 |
| *Primates* | *Mandrillus sphinx* | | 0.0116 | 2 | 16685.1 |
| *Carnivora* | *Panthera pardus* | | 0.0114 | 2 | 52400.0 |
| *Perisodactyla* | *Equus quagga* | | 0.0112 | 2 | 400000.0 |
| *Primates* | *Pan troglodytes schweinfurthii* | | 0.0107 | 2 | 45000.0 |
| *Carnivora* | *Genetta piscivora* | | 0.0090 | 2 | 1648.1 |
| *Carnivora* | *Profelis aurata* | | 0.0086 | 2 | 11277.2 |
| *Artiodactyla* | *Tragelaphus eurycerus* | | 0.0086 | 2 | 270998.4 |
| *Primates* | *Cercopithecus pogonias nigripes* | | 0.0082 | 2 | 3578.3 |
| *Carnivora* | *Bdeogale nigripes* | | 0.0082 | 2 | 2623.0 |
| *Primates* | *Pan troglodytes troglodytes* | | 0.0079 | 2 | 45000.0 |
| *Rodentia* | *Heliosciurus ruwenzorii* | | 0.0077 | 2 | 291.0 |
| *Primates* | *Allochrocebus lhoesti* | | 0.0068 | 2 | 4800.0 |
| *Artiodactyla* | *Neotragus batesi batesi* | | 0.0063 | 2 | 2967.5 |
| *Primates* | *Colobus angolensis cottoni* | | 0.0061 | 2 | 8990.3 |
| *Primates* | *Cercopithecus hamlyni* | | 0.0059 | 2 | 4550.0 |
| *Primates* | *Cercocebus chrysogaster* | | 0.0058 | 2 | 10300.0 |
| *Artiodactyla* | *Cephalophus ogilbyi crusalbum* | | 0.0058 | 1 | 18386.4 |
| *Primates* | *Pan paniscus* | | 0.0053 | 1 | 35120.0 |
| *Primates* | *Euoticus pallidus talboti* | | 0.0052 | 1 | 277.4 |
| *Lagomorpha* | *Poelagus marjorita* | | 0.0052 | 1 | 2509.7 |
| *Rodentia* | *Funisciurus carruthersi* | | 0.0051 | 1 | 276.9 |
| *Primates* | *Cercopithecus mitis mitis* | | 0.0051 | 1 | 5041.3 |
| *Primates* | *Galago matschiei* | | 0.0050 | 1 | 211.6 |
| *Primates* | *Sciurocheirus alleni cameronensis* | | 0.0050 | 1 | 360.0 |
| *Carnivora* | *Genetta poensis* | | 0.0045 | 1 | 3100.0 |
| *Primates* | *Lophocebus opdenboschi* | | 0.0041 | 1 | 6510.4 |
| *Carnivora* | *Genetta cristata* | | 0.0039 | 1 | 1863.2 |
| *Primates* | *Cercopithecus pogonias pogonias* | | 0.0037 | 1 | 3578.3 |
| *Proboscidea* | *Loxodonta cyclotis* | | 0.0034 | 1 | 4750000.0 |
| *Primates* | *Arctocebus calabarensis* | | 0.0034 | 1 | 258.0 |
| *Afrosoricida* | *Micropotamogale ruwenzorii* | | 0.0033 | 1 | 109.1 |
| *Primates* | *Procolobus rufomitratus lulindicus* | | 0.0029 | 1 | 8675.0 |
| *Primates* | *Cercopithecus erythrotis camerunensis* | | 0.0029 | 1 | 3540.2 |
| *Primates* | *Cercopithecus mitis heymansi* | | 0.0025 | 1 | 5041.3 |
| *Carnivora* | *Bdeogale jacksoni* | | 0.0023 | 1 | 2500.0 |
| *Artiodactyla* | *Neotragus batesi harrisoni* | | 0.0022 | 1 | 2967.5 |
| *Primates* | *Procolobus rufomitratus langi* | | 0.0021 | 1 | 8675.0 |
| *Primates* | *Cercopithecus mitis doggeti* | | 0.0021 | 1 | 5041.3 |
| *Carnivora* | *Genetta victoriae* | | 0.0021 | 1 | 2743.7 |
| *Artiodactyla* | *Cephalophus ogilbyi ogilbyi* | | 0.0020 | 1 | 18386.4 |
| *Artiodactyla* | *Okapia johnstoni* | | 0.0020 | 1 | 230001.1 |
| *Primates* | *Cercopithecus wolfi pyrogaster* | | 0.0020 | 1 | 3255.2 |
| *Primates* | *Pan troglodytes ellioti* | | 0.0019 | 1 | 45000.0 |
| *Primates* | *Colobus guereza matschiei* | | 0.0018 | 1 | 9925.9 |
| *Primates* | *Colobus angolensis cordeiri* | | 0.0018 | 1 | 8990.3 |
| *Primates* | *Cercopithecus ascanius atrinasus* | | 0.0017 | 1 | 3540.2 |
| *Primates* | *Cercopithecus wolfi elegans* | | 0.0015 | 1 | 3255.2 |
| *Artiodactyla* | *Tragelaphus derbianus* | | 0.0013 | 1 | 662500.0 |
| *Primates* | *Cercopithecus sclateri* | | 0.0013 | 1 | 3067.3 |
| *Primates* | *Lophocebus ugandae* | | 0.0010 | 1 | 7418.7 |
| *Primates* | *Mandrillus leucophaeus leucophaeus* | | 0.0010 | 1 | 14253.3 |
| *Primates* | *Procolobus rufomitratus foai* | | 0.0008 | 1 | 8675.0 |
| *Primates* | *Cercopithecus lomamiensis* | | 0.0006 | 1 | 5550.0 |
| *Rodentia* | *Paraxerus cooperi* | | 0.0006 | 1 | 250.0 |
| *Primates* | *Procolobus rufomitratus parmentieri* | | 0.0006 | 1 | 8675.0 |
| *Primates* | *Procolobus rufomitratus ellioti* | | 0.0006 | 1 | 8675.0 |
| *Primates* | *Gorilla beringei graueri* | | 0.0005 | 1 | 149325.2 |
| *Primates* | *Allochrocebus preussi pre* | | 0.0005 | 1 | 4500.0 |
| *Primates* | *Allochrocebus solatus* | | 0.0004 | 1 | 5450.0 |
| *Primates* | *Colobus angolensis ruwenzorii* | | 0.0003 | 1 | 8990.3 |
| *Rodentia* | *Funisciurus duchaillui* | | 0.0002 | 1 | 200.0 |
| *Primates* | *Procolobus rufomitratus tephrosceles* | | 0.0001 | 1 | 8675.0 |
| *Primates* | *Sciurocheirus alleni alleni* | | 0.0001 | 1 | 360.0 |
| *Primates* | *Euoticus pallidus pallidus* | | 0.0001 | 1 | 277.4 |
| *Primates* | *Lophocebus osmani* | | 0.0001 | 1 | 7418.7 |
| *Primates* | *Cercopithecus erythrotis erythrotis* | | 0.0001 | 1 | 3540.2 |
| *Primates* | *Colobus satanas satanas* | | 0.0000 | 1 | 9055.3 |
| *Primates* | *Colobus angolensis prigoginei* | | 0.0000 | 1 | 8990.3 |
| *Primates* | *Mandrillus leucophaeus poensis* | | 0.0000 | 1 | 14253.3 |
| *Primates* | *Allochrocebus preussi ins* | | 0.0000 | 1 | 4500.0 |
| *Primates* | *Procolobus pennantii pennantii* | | 0.0000 | 1 | 10500.0 |
| *Primates* | *Procolobus preussi* | | 0.0000 | 1 | 7300.0 |
| *Primates* | *Procolobus pennantii bouvieri* | | 0.0000 | 1 | 10500.0 |
| *Primates* | *Cercopithecus dryas* | | 0.0000 | 1 | 2784.1 |
| *Primates* | *Cercopithecus mitis schoutedeni* | | 0.0000 | 1 | 5041.3 |
| *Primates* | *Gorilla gorilla diehli* | | 0.0000 | 1 | 112589.0 |
| *Primates* | *Gorilla gorilla gorilla* | | 0.0000 | 1 | 112589.0 |
| *Primates* | *Gorilla beringei beringei* | | 0.0000 | 1 | 149325.2 |
| *Perissodactyla* | *Diceros bicornis* | | 0.0000 | 1 | 995940.5 |

* ^1^Fa *et al*. (2014)

** ^2^Jones et al. (2009); ^3^Hart et al. (2012); ^4^ Kingdon et al. (2013).

**Appendix S2:** Hypothesis testing when domestic meat is included in the models.

**Table S1.** **Standardized weights (SW) and statistical significance (*P*) of regressions.** Hypotheses tested for the relationship between mammal diversity and stunting: ***H*_1_** (direct relationship); ***H*_2_** (diversity influences human variables, and these influence stunting); ***H*_3_** (human variables influence both diversity and stunting). **DRD**: Deep Rainforest Diversity; **MRD**: Marginal Rainforest Diversity. To identify dependent and independent variables, see Fig. S1.

|  | **Diversity** | | **Stunting prevalence** | |
| --- | --- | --- | --- | --- |
|  | **SW** | ***P*** | **SW** | ***P*** |
| H_2_ **-DRD** |  |  |  |  |
| **DRD** |  |  | -0.028 | 0.873 |
| **Rural population density** | -0.034 | 0.001 | 5.381 | 0.007 |
| **Distance from urban areas** | 0.246 | 0.066 | -0.291 | 0.066 |
| **Distance from roads** | 0.466 | <0.001 | <0.001 | 0.907 |
| **Distance from protected areas** | -1.199 | 0.067 | 0.079 | 0.010 |
| **Domestic meat** | -7.123 | 0.341 | 0.003 | 0.278 |
| H_3_ **- DRD** |  |  |  |  |
| **DRD** |  |  | 0.028 | 0.8790 |
| **Rural population density** | -4.646 | <0.001 | 5.381 | 0.007 |
| **Distance from urban areas** | -0.105 | 0.344 | -0.291 | 0.157 |
| **Distance from roads** | <0.001 | <0.001 | <0.001 | <0.001 |
| **Distance from protected areas** | -0.069 | <0.001 | 0.079 | 0.011 |
| **Domestic meat** | -0.004 | 0.011 | 0.003 | 0.299 |
| H_2_ **- MRD** |  |  |  |  |
| **MRD** |  |  | 1.132 | 0.387 |
| **Rural population density** | 0.372 | <0.001 | 3.961 | 0.084 |
| **Distance from urban areas** | -0.717 | 0.432 | -0.333 | 0.038 |
| **Distance from roads** | -2.719 | <0.001 | <0.001 | 0.839 |
| **Distance from protected areas** | 0.642 | 0.886 | 0.069 | 0.019 |
| **Domestic meat** | -55.305 | 0.267 | 0.002 | 0.309 |
| H_3_ **- MRD** |  |  |  |  |
| **MRD** |  |  | 1.132 | 0.387 |
| **Rural population density** | 1.140 | <0.001 | 3.961 | 0.091 |
| **Distance from urban areas** | 0.034 | 0.029 | -0.333 | 0.039 |
| **Distance from roads** | <0.001 | 0.017 | <0.001 | 0.840 |
| **Distance from protected areas** | 0.007 | 0.011 | 0.069 | 0.019 |
| **Domestic meat** | <0.001 | 0.854 | 0.002 | 0.304 |

**Table S2.** **Fit summary for models relating mammal diversity, stunting and human pressure.** **χ^2^**: test of differences between observed and expected covariance matrices; **P**: statistical significance of **χ**^2^; **TLI**: Tucker-Lewis Index; **CFI**: Comparative Fit Index; **NFI**: Normed Fit Index; **RMSEA**: Root Mean Square Error of Approximation; **AIC**: Akaike Information Criterion. Hypotheses tested for the relationship between diversity and stunting: ***H*_2_** (diversity influences human variables, and these influence stunting); ***H*_3_** (human variables influence both diversity and stunting). **DRD**: Deep Rainforest Diversity; **MRD**: Marginal Rainforest Diversity. All the statistics showed the best fit of *H_2_* for **DRD**, and of *H_3_* for **MRD**.

|  | **χ^2^** | ***P*_[d.f.=7]_** | **TLI** | **CFI** | **NFI** | **RMSEA** | **AIC** |
| --- | --- | --- | --- | --- | --- | --- | --- |
| H_2_ **- DRD** | 11.892 | 0.104 | 0.824 | 0.941 | 0.886 | 0.110 | 67.892 |
| H_3_ **- DRD** | 8.074 | 0.326 | 0.961 | 0.987 | .923 | 0.061 | 64.074 |
| H_2_ **- MRD** | 7.218 | 0.407 | 0.993 | 0.998 | 0.936 | 0.023 | 63.218. |
| H_3_ **- MRD** | 8.074 | 0.326 | 0.965 | 0.988 | 0.928 | 0.051 | 64.074 |

**Figure S1. Path diagrams representing relationships between diversity and stunting among children** **considering domestic meat as a factor.** Hypotheses tested: H_2_ (diversity influences human variables, and these influence stunting); H_3_ (human variables influence both diversity and stunting). (**A**) Models for Deep Rainforest Diversity (DRD). (**B**) Models for Marginal Rainforest Diversity (MRD). Circles enclosing "e": error terms associated to dependent variables. Solid black arrows: significant relationships (*n* = 60; *P* < 0.05); dashed black arrows: 0.05 < *P* < 0.07; grey arrows: non-significant relationships (*P* > 0.07); double arrows: covariance between variables, which are considered in the diagrams when significant correlations were identified within the study area (*P* < 0.05). +: Positive relationship; - : Negative relationship.


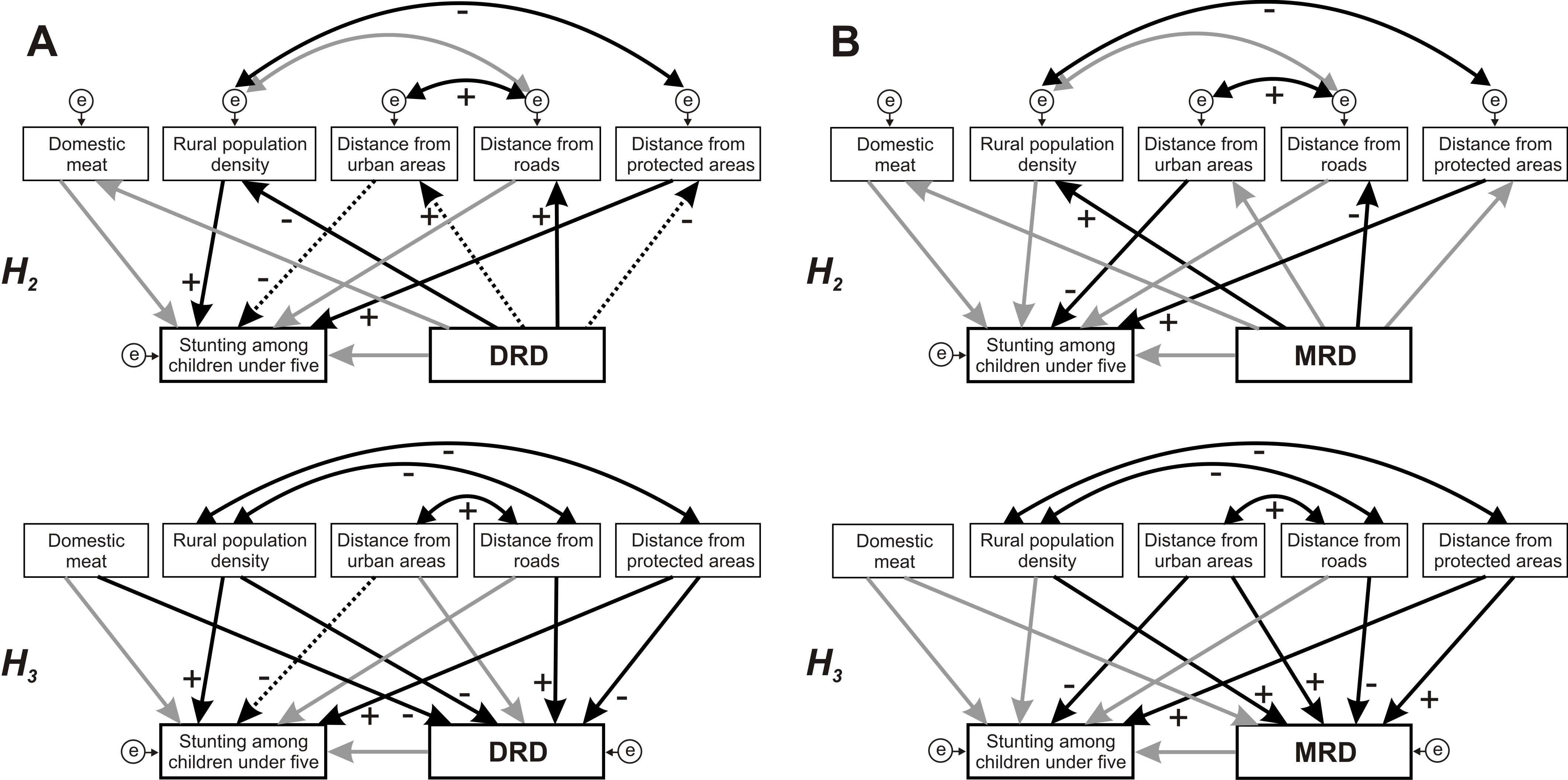


**Appendix S3. Hypothesis testing. In this table we present the reasoning behind the observed significant correlations of stunting among children and diversity. Our hypotheses are theoretical proposals that we make compete with each other for consistence, and are built on the evidence described in explanatory notes below.**

|  |  | **DRD areas** |  | **MRD areas** |  |
| --- | --- | --- | --- | --- | --- |
|  |  | Hypothesis | Expected if true | Hypothesis | Expected if true |
|  | H_1_ | As bushmeat in deep forests is the main source of animal-based nutrients [1], and diversity correlates with wild meat availability [2], a higher DRD results in better food security. | The direct negative relationship between DRD and stunting remains significant when alternative explanatory paths are considered within the model. | A direct cause-effect hypothesis explaining the negative relation between MRD and stunting would mean that bushmeat fails to assure nutritional needs of people subsisting from hunting. | The direct positive relationship between MRD and stunting remains significant when alternative explanatory paths are considered within the model. |
|  | H_2_ | Although evidences indicate that external hunters are penetrating deep forests [3], the remoteness of DRD areas still prevents the establishment of dense human populations [4]. A high prevalence of stunting would be associated to the socio-economy of densely populated areas [5]. | Areas of high DRD (deep forest) are significantly described by the remoteness of roads and populated areas. Stunting prevalence is, in turn, explained by the proximity of densely populated areas. | The demand for wild meat at all levels of the African society turns areas of high bushmeat production into attractive sites for human settlements [6]. As population increases [7], demands for bushmeat grows over greater distances [8]. Socio-economic drivers of stunting [5] would make malnutrition reach forest margins as they are occupied and transformed. | High MRD significantly explains the proximity of roads and populated places. Stunting prevalence is, in turn, explained by the proximity of densely populated areas. |
|  | H_3_ | High stunting prevalence would be associated with the socioeconomic conditions of densely populated areas [5]. Simultaneously, population growth resulting from higher exploitation of forests increases demand for wild meat [9], resulting in a decline of DRD. | Indicators of human population density predict low DRD, whereas wildlife protection is associated with higher DRD. Stunting prevalence is, in turn, explained by proximity to densely populated areas. | MRD species, mostly smaller, fast-reproducing and high-density species [10], can find conditions of high hunting pressure favorable and densities may even increase [11,12,13]. Demand for hunting is, however, substantially increased by urban populations [8]. An increase of MRD is consistent with the existence of a medium-scale disturbance derived from human pressure [14]. A high prevalence of stunting could also be associated with socio-economic conditions of densely populated areas [5]. | High rural populations and lack of protection against hunting predict a high MRD, whereas the proximity of urban areas makes MRD decrease. Stunting prevalence is, in turn, explained by the proximity of densely populated areas. |

Explanatory Notes:

1. Bushmeat in remote forest areas is an important source of protein and other nutrients for many inhabitants ^5, 6^. Drivers of hunting are the need for wild meat as an important food, as well as a source of income ^7, 8, 9^.
2. There is significant overlap of the distribution patterns followed by standing biomass and diversity, in both DRD and MRD (results in this report).
3. Ecological survey results suggest that hunters penetrate up to 40 km from roads and rivers into deep forest^10^. With projected high human population growth rates, hunting offtake in central African forests is expected to increase and spread into the last remnants of remote forest ^10^.
4. Forest areas in central Africa are considered to be among the most remote areas in the world, in terms of density of human settlements and infrastructures^11^.
5. Human nutritional status is affected by the interplay of socio-economic and demographic factors ^12^.
6. Wild meat is a traditional food, consumed by all socio-economic sectors in central Africa ^13,14^. The availability of this free resource encourages users across the region ^8,15^.
7. Deforestation in central Africa is mostly related to increases in human population density and subsequent land conversion for agriculture^16^.
8. With greater urbanization trends of African societies, the demand for bushmeat is expected to increase^17^. Large and growing urban centers create substantial demand for natural resources over forest areas often hundreds of kilometers away^18^.
9. Logging operations and other extractive industries (e.g. mining ^19^) concentrate and expand population growth, and rising incomes increase demand for wild meat and hunting pressure ^20, 21^.
10. MRD species are mostly small, fast-reproducing and high-density generalist species, such as rodents, small carnivores and galagos^1^.
11. Large, low-density, slow-reproducing and specialist species are more vulnerable to increases in hunting pressure than smaller, fast-reproducing and high-density generalist species ^22, 23^.
12. Small species may find conditions of high hunting pressure favorable when their natural predators and competitors are hunted to low densities^24,10, 25^.
13. Overhunting can cause trophic cascade changes, only central African forests still harbor significant populations of large mammals^10^.
14. The intermediate-disturbance hypothesis^26, 27^ postulate that diversity is higher at intermediate intensities of disturbance, when competitive exclusion and loss of competitive dominants are balanced. Low levels of disturbance allow better competitors to persist, whereas few species can persist when disturbances are very intense.

**Appendix S4**

**Variable Sources**

1. Prevalence of stunting among children under 5 years of age: reported on 06/25/2007 by FAO according to the lowest available sub-national administrative units, compiled from Demographic and Health Surveys (DHS), UNICEF MICS, WHO Global Database on Child Growth and Malnutrition, and national surveys ^28^.
2. Rural human population density: estimated by combining LandScan 2008™ High Resolution Global Population Data Set (copyrighted by UT-Battelle, LLC, operator of Oak Ridge National Laboratory) with MODIS 500-m Map of Global Urban Extent^29,30^.
3. Distance from urban areas: derived from the Administrative Centres & Populated Places shapefile at the Relational World Database II (RWDB2) ([http://data.fao.org](http://data.fao.org/)).
4. Distance from roads: calculated using the maps extracted by FAO/GIS (http://www.fao.org/geonetwork) from Vector Map Level 0 at the Digital Chart of the World (DCW, [http://worldmap.harvard.edu](http://worldmap.harvard.edu/)).
5. Distance from protected areas: the network recorded at the World Database on Protected Areas (WDPA)^31^ has been used as a reference.
6. Domestic meat: we have used estimates of supply and demand for animal-source foods provided by FAO^32^ (2011). Food balances were computed as the difference between production (considering beef, mutton, pork and poultry together) and consumption (kg/km^2^).

**References**

1. Fa, J. E. *et al*. Integrating sustainable hunting in biodiversity protection in central Africa: hot spots, weak spots, and strong spots. *PLoS ONE* 9(11): e112367. doi: 10.1371/journal.pone.0112367 (2014).
2. Jones, K. E. *et al*. PanTHERIA: a species-level database of life history, ecology, and geography of extant and recently extinct mammals. *Ecology* **90**, 2648 (2009).
3. Hart, J.A., Detwiler, K.M., Gilbert, C.C., Burrell, A.S., Fuller, J.L., et al. Lesula: A new species of *Cercopithecus* monkey endemic to the Democratic Republic of Congo and implications for conservation of Congo’s Central Basin. *PLoS ONE* 7(9): e44271. doi:10.1371/journal.pone.0044271 (2012).
4. Kingdon J. *et al*. (Eds). *Mammals of Africa: 6 Vols*. (Bloomsbury Publishing London, 2013).
5. Nasi, R., Taber, A. & Van Vliet, N. Empty forests, empty stomachs? Bushmeat and livelihoods in the Congo and Amazon Basins. *Int Forest Rev* **13**, 355-368 (2011).
6. Golden, C. et al. Benefits of wildlife consumption to child nutrition in a biodiversity hotspot. *P Natl Acad Sci USA* **108**, 19653-19656 (2011).
7. Brashares, J. S. *et al*. Economic and geographic drivers of wildlife consumption in rural Africa. *P Natl Acad Sci USA* **108**, 13931-13936 (2011).
8. Kümpel, N. F., Milner-Gulland, E. J., Cowlishaw, G. & Rowcliffe, J. M. Incentives for hunting: the role of bushmeat in the household economy in rural Equatorial Guinea. *Hum Ecol* **38**, 251–264 (2010).
9. Foerster S. *et al*. Correlates of bushmeat hunting among remote rural households in Gabon, Central Africa. *Conserv Biol* **26**, 335–344 (2012).
10. Abernethy, K. A. *et al*. Extent and ecological consequences of hunting in Central African rainforests in the twenty-first century. *Philos T Roy Soc B* **368**, 20120303 (2013).
11. Wildlife Conservation Society - WCS, and Center for International Earth Science Information Network - CIESIN - Columbia University. *Last of the Wild Project*, Version 2, 2005 (LWP-2): Global Human Footprint Dataset (IGHP). Palisades, NY: NASA Socioeconomic Data and Applications Center (SEDAC) (2005). http://dx.doi.org/10.7927/H4M61H5F (accessed 4 December 2014).
12. Yimer, G. Malnutrition among children in Southern Ethiopia: Levels and risk factors. *Ethiopian J Health Develop* **14.3**, 283-292 (2000).
13. Wilkie, D. S. *et al*. Role of prices and wealth in consumer demand for bushmeat in Gabon, Central Africa. *Conserv Biol* **19**, 268–274 (2005).
14. Nasi, R. et al. *Conservation and Use of Wildlife-Based Resources: The Bushmeat Crisis*. Convention on Biological Diversity, Montreal, Canada, and Center for International Forestry Research, Bogor, Indonesia (2008).
15. de Merode, E., Homewood, K. & Cowlishaw, G. The value of bushmeat and other wild foods to rural households living in extreme poverty in Democratic Republic of Congo. *Biol Conserv* **118**, 573–581 (2004).
16. Mayaux, P. *et al*. State and evolution of the African rainforests between 1990 and 2010*. Philos T Roy Soc B* **368**, 20120300 (2013).
17. Ziegler, S. Application of food balance sheets to assess the scale of the bushmeat trade in Central Africa. *TRAFFIC Bull* **22**, 1-12 (2011).
18. Ape Alliance. *The African Bushmeat Trade—A Recipe for Extinction*. Cambridge, UK, (1998).
19. Edwards, D. P. *et al*. Mining and the African environment. *Conserv Lett* **7**, 302–311 (2014).
20. Poulsen, J. R., Clark, C. J., Mavah, G. & Elkan, P. W. Bushmeat supply and consumption in a tropical logging concession in Northern Congo. *Conserv Biol* **23**, 1597–608 (2009).
21. Thibault, M. & Blaney S. The oil industry as an underlying factor in the bushmeat crisis in Central Africa. *Conserv Biol* **17**, 1807–0813 (2003).
22. Berryman, A. A. The origins and evolution of predator prey theory. *Ecology* **73**, 1530–1536 (1992).
23. Robinson, J. G. & Bennett, E. L. (editors) *Hunting for Sustainability in Tropical Forests*. (Columbia University Press, New York, 2000).
24. Peres, C. A. & Palacios, E. Basin-wide effects of game harvest on vertebrate population densities in Amazonian forests: implications for animal mediated seed dispersal. *Biotropica* **39**, 304–315 (2007).
25. Effiom, O. A., Nunez-Iturri, G., Smith, H. G. & Olsson, O. Bushmeat hunting changes regeneration of African rainforests. *Proceedings of the Royal Society B* **280**, 20130246 (2013).
26. Connell, J. H. Diversity in tropical rain forests and coral reefs. *Science* **199**, 1302–1310 (1978).
27. Huston, M. A. *Biological Diversity: The Coexistence of Species on Changing Landscape*s. (Cambridge University Press, Cambridge UK, 1994).
28. FAO*. Chronic Undernutrition among Children: An Indicator of Poverty*. FAO/SDRN & ESNA, Rome (2003).
29. Schneider, A., Friedl, M. A. & Potere, D. A new map of global urban extent from MODIS data. *Environ Res Lett* **4**, 044003 (2009).
30. Schneider, A., Friedl, M. A. & Potere, D. Monitoring urban areas globally using MODIS 500m data: New methods and datasets based on urban ecoregions. *Remote Sens Environ* **114**, 1733-1746 (2010).
31. UNEP-WCMC. *Data Standards for the World Database on Protected Areas*. UNEP-WCMC, Cambridge (2012).
32. FAO. *Mapping Supply and Demand for Animal-Source Foods to 2030*. Animal Production and Health Working Paper, No, 2. (FAO, Rome, 2011).
